# Symptom profiling for infectious intestinal disease (IID): a secondary data analysis of the IID2 study

**DOI:** 10.1017/S0950268819001201

**Published:** 2019-06-28

**Authors:** A. L. Donaldson, H. E. Clough, S. J. O'Brien, J. P. Harris

**Affiliations:** 1 NIHR Health Protection Research Unit in Gastrointestinal Infections, University of Liverpool, Liverpool, UK; 2 Institute of Population Health Sciences, University of Liverpool, Liverpool, UK

**Keywords:** Epidemiology, Gastroenteritis, Infectious intestinal disease, Surveillance, Symptomology

## Abstract

Less than half of stool samples from people symptomatic with infectious intestinal disease (IID) will identify a causative organism. A secondary data analysis was undertaken to explore whether symptomology alone could be used to make inferences about causative organisms. Data were utilised from the Second Study of Infectious Intestinal Disease in the Community. A total of 844 cases were analysed. Few symptoms differentiated individual pathogens, but grouping pathogens together showed that viral IID was more likely when symptom onset was in winter (odds ratio (OR) 2.08, 95% confidence interval (CI) 1.16–3.75) or spring (OR 1.92, 95% CI 1.11–3.33), the patient was aged under 5 years (OR 3.63, 95% CI 2.24–6.03) and there was loss of appetite (OR 2.19, 95% CI 1.29–3.72). The odds of bacterial IID were higher with diarrhoea in the absence of vomiting (OR 3.54, 95% CI 2.37–5.32), diarrhoea which persisted for >3 days (OR 2.69, 95% CI 1.82–3.99), bloody diarrhoea (OR 4.17, 95% CI 1.63–11.83) and fever (OR 1.67, 95% CI 1.11–2.53). Symptom profiles could be of value to help guide clinicians and public health professionals in the management of IID, in the absence of microbiological confirmation.

## Introduction

Infectious intestinal disease (IID) is characterised by the acute onset of diarrhoea and/or vomiting in otherwise healthy people caused by an infectious, transmissible organism [1]. In the UK, the surveillance of IID is based on statutory notifications, outbreak reports and syndromic surveillance from primary, secondary and remote health services [2, 3]. However, as the syndrome of diarrhoea and vomiting can have non-infectious causes, microbiological confirmation remains central to conclusive diagnosis of IID. Although microbiological testing is the gold standard, in some cohort studies, causative organisms have only been identified in 37–46% of samples from symptomatic individuals [1, 4, 5]. The likelihood of identifying a causative organism has been found to be affected by factors such as age, sex, occupation, the absence of specific symptoms such as vomiting, and the timing of the stool sample in relation to symptom onset [1]. Other factors such as the volume of the sample, the performance of the microbiological test and local organism testing policies may also impact on the isolation of organisms [6, 7].

This diagnostic gap means that for over half of symptomatic patients, the cause of illness will not be identified. Whilst the majority of IID cases are self-limiting, being aware of the underlying cause can be of value in both case and outbreak management. For outbreak situations, epidemiological criteria have been developed which utilise, among other factors, the proportion of people affected by given symptoms in order to make inferences as to the underlying organism. The most notable of these is the Kaplan criteria [8] which were developed in the 1980s in response to the lack of diagnostic tests available for isolating norovirus. Kaplan identified that, where no bacterial organism had been identified in stool cultures, outbreaks were more likely to be caused by norovirus when >50% of people were affected by vomiting; the incubation period was 24–48 h; and the mean duration of illness was 12–60 h. A subsequent re-evaluation of these criteria, once diagnostic tests became available, found them to be highly specific (99%) and moderately sensitive (68%) at distinguishing outbreaks of norovirus from bacterial IID outbreaks [9]. Other epidemiological criteria have also been proposed, including a greater fever–vomiting ratio [10] and a higher diarrhoea–vomiting ratio in bacterial outbreaks, suggesting that fever and diarrhoea are more indicative of a bacterial cause [11]. However, the basis of epidemiological criteria is the relative prevalence of symptoms occurring within a group of affected people, and as such they cannot be applied to individual cases. Seasonal outbreaks of IID may present as an increase in reporting of individual cases and therefore being able to ascribe likely cause to single cases of IID has public health and epidemiological value, as well as clinical application. This study uses data from a large community cohort and General Practice study to investigate whether symptoms alone can be used to make inferences as to the causative organisms for individual cases of IID.

## Methods

### Data sources

A secondary data analysis was undertaken using data from the Second Study of Infectious Intestinal Disease in the Community (IID2 Study), the methodology of which is detailed elsewhere [1, 12]. This analysis included data from the two main components of the IID2 study: the General Practice (GP) presentation study, which was a 12-month prospective study of people consulting a GP with symptoms of IID; and the prospective population-based cohort study, which involved weekly follow-up of healthy volunteers in the community to identify any symptoms of IID. The case definition for IID that was used in the original study was loose stools or clinically significant vomiting lasting <2 weeks, in the absence of a known non-infectious cause. Both studies utilised symptom questionnaires and stool sample testing of symptomatic people who met the case definition. Cases were included in this analysis if they had completed a symptom questionnaire and submitted a stool sample. Cases with negative stool samples, where no pathogen was identified, were excluded. Data from dual and triple infections were included multiple times; once for each organism identified, as the primary cause of symptoms could not be determined.

### Data analysis

Multivariable logistic regression was used to determine the odds of a case being caused by a given pathogen based on reported symptoms. The explanatory variables included the symptoms outlined in the IID2 study symptom questionnaire, along with the participant's age and date of symptom onset (Table 1). Continuous data, namely symptom duration, date of illness onset and age, were categorised before inclusion in the regression models. Given that diarrhoea and vomiting are the predominant symptoms of IID and many people will have both, variables were created to capture cases of diarrhoea in the absence of vomiting, and vomiting in the absence of diarrhoea. These variables were used to explore whether this is a symptom profile which offers discrimination between pathogens. Phi coefficients were used to identify any significant correlations between the explanatory variables which might lead to mathematical problems with model fitting.

**Table 1.** Explanatory variables included in the multivariate analysis and their coding

Explanatory variables	Coding
*Symptoms from IID2 questionnaire^a^*	
Diarrhoea days	>3 days = 1 ⩽3 days = 0
Bloody diarrhoea	Yes = 1 No = 0
Vomiting days	>3 days = 1 ⩽3 days = 0
Nausea	Yes = 1 No = 0
Nausea days	>3 days = 1 ⩽3 days = 0
Abdominal pain	Yes = 1 No = 0
Loss of appetite	Yes = 1 No = 0
Fever	Yes = 1 No = 0
Headache	Yes = 1 No = 0
Cough/nose/throat	Yes = 1 No = 0
Combined symptom variables	
Diarrhoea but no vomiting	Yes = 1 No = 0
Vomiting but no diarrhoea	Yes = 1 No = 0
*Participant characteristics*	
Age^b^	>16 years = 0, 5–16 years = 1, <5 years = 2
Date of onset^b^	Autumn(0), Winter(1), Spring(2), Summer(3)^c^

a ‘Not sure’ responses from the original questionnaires were left blank and treated as missing data.

b Coded as factors for analysis.

c Seasons defined by meteorological calendar.

The outcome variable was the presence of the infectious organism. Pathogens which accounted for >10% of the total number of cases were analysed independently, to identify symptoms which distinguished them from any other cause of IID. Below this threshold, case numbers were too small to generate meaningful output for a single organism. Grouped organism models were used to capture differences between the broader classes of pathogen; bacteria, viruses and protozoa, sequentially comparing one class against any other cause of IID.

Statistical analysis was undertaken in R 3.3.2 [13]. Odds ratios (OR) were calculated using binomial backward stepwise regression. Models were selected based on the Akaike information criterion (AIC). Upper and lower 95% confidence intervals (CI) were calculated around each estimate.

## Results

There was a total of 1657 cases identified from the IID2 study which met the IID2 case definition and had both completed a questionnaire and submitted a stool sample. Of these, 898 cases (54%) were excluded from the analysis as no organism was identified from their stool sample. The total sample size for analysis was 844; including 69 dual infections and eight triple infections.

Norovirus was the most commonly identified cause of IID, and campylobacter was the commonest bacterial cause (Table 2). Only four pathogens met the criteria for organism-specific analysis; norovirus, campylobacter, rotavirus and sapovirus. The total number of protozoal infections was <10% of the total number of cases and consequently grouped organism models were only generated for bacterial and viral IID. To capture any important differences in symptoms, protozoa were included in the comparison group for both the bacterial and viral models.

**Table 2.** Organisms and the associated number of cases, as included in the analysis

Pathogen	No. cases identified
Bacteria	238
*C. difficile*	11
*C. perfringens*	25
*Campylobacter* sp.	150
*E.coli* VTEC	15
Enteroaggregative *E.coli*	27
*Salmonella* sp.	9
*Yersinia* sp.	1
Viruses	576
Adenovirus	58
Astrovirus	36
Norovirus	237
Rotavirus	96
Sapovirus	149
Protozoa	30
*Cryptosporidium* sp.	15
*Giardia* sp.	15
Total organisms identified	844

The grouped organism models (Table 3) showed that the odds of the causative organism being bacterial were higher with diarrhoea in the absence of vomiting (OR 3.54, 95% CI 2.37–5.32), diarrhoea which persisted for >3 days (OR 2.69, 95% CI 1.82–3.99), bloody diarrhoea (OR 4.17, 95% CI 1.63–11.83) and fever (OR 1.67, 95% CI 1.11–2.53). The odds of a viral cause of illness were higher when symptom onset was in winter (OR 2.08, 95% CI 1.16–3.75) or spring (OR 1.92, 95% CI 1.11–3.33), the patient was under 5 years of age (OR 3.63, 95% CI 2.24–6.03) and there was loss of appetite (OR 2.19, 95% CI 1.29–3.72). Given protozoa have a similar aetiology to bacterial IID, as contrasted to viral IID, the analysis was repeated with protozoa assigned to the bacterial group to explore what impact this would have on the symptom profiling. The resulting viral and bacterial/protozoal models did not differ significantly from the above models; the same explanatory variables were identified, but the significance of winter and spring in the bacteria/protozoa model was increased.

**Table 3.** Grouped organism multivariate model outputs (OR with 95% confidence intervals) for bacterial and viral pathogens, as compared to any other pathogen

Explanatory variable	Odds ratio (95% confidence intervals)	
	Bacterial	Viral
Aged <5 years	0.25 (0.14–0.41)	3.63 (2.24–6.03)
Onset in winter	0.65 (0.35–1.19)	2.08 (1.16–3.75)
Onset in spring	0.70 (0.40–1.23)	1.92 (1.11–3.33)
Diarrhoea but no vomiting	3.54 (2.37–5.32)	0.27 (0.18–0.39)
Bloody diarrhoea	4.17 (1.63–11.83)	0.30 (0.11–0.79)
Diarrhoea lasting >3 days	2.69 (1.82–3.99)	0.33 (0.22–0.48)
Loss of appetite	0.44 (0.26–0.75)	2.19 (1.29–3.72)
Fever	1.67 (1.11–2.53)	0.59 (0.39–0.88)

The organism-specific modelling generated less meaningful outputs. The campylobacter model largely mirrored the grouped bacterial model and did not provide any further discriminatory information. The virus-specific analysis for norovirus, rotavirus and sapovirus was sensitive to changes in the parameters of the models which led to inconsistent symptom profiles. Phi coefficients were used to identify any significant correlations between the binary explanatory variables which could impact on the model fitting. There was no evidence of significant co-linearity which would affect the modelling, although there were some mild-to-moderate correlations (phi coefficient <0.5) between some symptoms such as nausea and loss of appetite.

## Discussion

This study has identified that people with IID who reported symptoms of diarrhoea in the absence of vomiting, diarrhoea lasting for more 3 days, bloody diarrhoea and fever were at increased odds of having a bacterial pathogen. Young age (<5 years), onset in spring or winter and loss of appetite were associated with increased odds of viral cause. These findings are consistent with other studies which have found associations between bacterial pathogens and symptoms of fever, bloody diarrhoea and prolonged illness, whilst vomiting and a short duration of symptoms have been associated with viral causes [14–17]. Epidemiological criteria, utilised in outbreak situations, have similarly highlighted the importance of vomiting and short duration of illness as indicative of norovirus, whilst symptoms such as fever and diarrhoea have been associated with bacterial outbreaks [8–11]. This study is largely consistent with these criteria, identifying similar associations between these symptoms and the class of the underlying pathogen. However, our analysis would suggest that vomiting and a short duration of symptoms are better ascribed to viral IID than any single viral pathogen. Furthermore, the duration of norovirus symptoms is known to be affected by individual risk factors, such as hospitalisation and age [18, 19]. Therefore, the 12–60 h duration used in the Kaplan criteria may be less applicable when considering individual cases of norovirus illness. This study used ⩽3 days to categorise symptom duration, which provided good discrimination between bacterial and viral causes of IID.

This analysis did not identify symptoms which could be used to adequately differentiate individual IID pathogens. This should act as a caution against making assumptions about the underlying organism on the basis of symptoms alone. However, this dataset did not contain sufficient numbers of some organisms to generate the statistical power necessary to model at the level of individual pathogens. Furthermore, the mild-to-moderate correlations identified between certain symptoms could make it harder for statistical models to distinguish individual pathogens on the basis of these symptoms alone.

The findings of this study have application for clinicians, public health professionals and epidemiologists, who use symptoms to generate hypotheses regarding causative organisms when managing cases and outbreaks of IID. This analysis would suggest that assumptions should not be made as to the individual pathogen in the absence of microbiological confirmation. However, given the different transmission patterns and natural histories of bacterial and viral IID [20], using symptom profiles to indicate a likely bacterial or viral cause could assist the early stages of outbreak investigations when microbiology is not yet available. This could help guide infection prevention and control; for example, viral causes are more likely to be spread person to person, whereas bacterial IID would raise suspicion of a food or animal contact. Given the large diagnostic gap for IID, the role of symptoms is still of vital importance to guide clinical and public health action. These findings could have further application for syndromic surveillance systems, enabling symptomatic cases to be categorised as either suspected bacterial or viral IID. However, the benefits of this would have to be weighed against the practical challenges of developing sensitive and specific case definitions that would be compatible with the level of symptom detail gathered and recorded by syndromic surveillance systems [21].

### Strengths and limitations

This study utilised data from a large prospective cohort study [12], removing some of the reporting biases inherent within national surveillance data [22]. However, given that the severity and duration of illness is known to affect health-seeking behaviour and stool sample submission [22], mild short-lived illness is still likely to be underrepresented in these data. Despite the large size of the dataset, the total numbers of some organisms were too low to allow organism-specific models to be developed for all but the four most common causes. Furthermore, protozoa could not be examined as a separate class of pathogen due to small numbers. In this analysis, protozoa were included in the comparison group for both the bacterial and viral models. To explore the impact this could have had on the modelling, the analysis was repeated with protozoa assigned to the bacterial group. The resulting viral and bacterial/protozoal models did not differ significantly from the original models indicating that the group allocation of the protozoa had little impact on the findings of this analysis.

It should be considered that the grouped organism profiles will be naturally weighted by the relative prevalence of different organisms within each class; campylobacter accounted for almost two-thirds of all the bacterial cases and norovirus accounted for over 40% of viral cases. Consequently, the bacterial and viral models will disproportionality reflect the symptoms associated with these pathogens. However, this reflects real-life diagnostics where certain symptoms or organisms are more likely simply because they occur more commonly. Whilst this analysis could not identify symptom profiles which discriminated individual pathogens, this is an area that warrants further exploration. Future studies could also consider the role of co-infections, as co-infections have been found to affect the pathogenicity of organisms [23].

## Conclusion

Symptom profiles could be used to help dissociate between bacterial and viral causes of IID however, symptoms do not allow further discrimination of individual organisms. Microbiology remains the gold standard and where possible, microbiological confirmation is recommended. However, in situations where microbiology is not available or results are inconclusive, symptom profiling could be of value for clinicians, public health professionals and epidemiologists to distinguish likely bacterial and viral pathogens to guide the management of cases and outbreaks of IID.
